# Clinical Characteristics and Outcomes of Patients Presenting with Acute Coronary Syndromes and Suspected Plaque Erosion Based on Clinical and Laboratory Criteria

**DOI:** 10.3390/jcdd12090335

**Published:** 2025-08-30

**Authors:** Luca Di Vito, Giancarla Scalone, Federico Di Giusto, Filippo Bruscoli, Michele Alfieri, Domenico Delfino, Federico Panzella, Simona Silenzi, Ik-Kyung Jang, Pierfrancesco Grossi

**Affiliations:** 1 Cardiology Unit, C. and G. Mazzoni Hospital, AST, 63100 Ascoli Piceno, Italy; gcarlascl@gmail.com (G.S.);; 2 Cardiology and Arrhythmology Clinic, Marche Polytechnic University of Ancona, 60126 Ancona, Italy; 3 Cardiology Division, Massachusetts General Hospital—Harvard Medical School, Boston, MA 02114, USA; ijang@mgh.harvard.edu

**Keywords:** plaque erosion, plaque rupture, acute coronary syndromes, optical coherence tomography

## Abstract

**Background:** Plaque erosion (PE) ranks as the second most prevalent pathology associated with acute coronary events, following plaque rupture. PE is characterized by endothelial denudation and the development of neutrophil extracellular traps. Specific clinical and laboratory predictors were shown to be associated with PE in patients with acute coronary syndrome. The aim of this study was to evaluate the clinical and laboratory results, as well as the outcomes of ACS patients with a high likelihood of PE. **Methods:** A total of 696 ACS patients were categorized into the suspected PE group and the less likely PE group based on the five validated predictors of PE. Baseline clinical characteristics and laboratory evaluations were analyzed between the two groups. Major adverse cardiovascular events were compared between the two groups at 20 months. **Results:** The group suspected of PE comprised 41% of patients, whereas the group with a lower likelihood of PE constituted 59%. The suspected PE group exhibited a greater incidence of current smokers and a higher BMI. Both CRP and fibrinogen levels were decreased; the incidence of one coronary vessel disease was elevated. The suspected PE group exhibited a markedly reduced incidence of MACEs at 20 months (7.4% compared to 28.8%, *p* = 0.0001). The recurrence of non-fatal coronary events tended to occur later in the suspected PE group (15 months (6–20) compared to 9 months (6–13), *p* = 0.062). A reduced coronary plaque burden and a low level of systemic inflammation characterized the distinctive features of the suspected PE cohort. **Conclusions:** The suspected PE group exhibited a more favorable prognosis at the 20-month follow-up, characterized by a considerably reduced mortality rate from all causes, whereas non-fatal coronary events tended to manifest at a later time.

## 1. Introduction

The second most common mechanism triggering an acute coronary syndrome (ACS) event after plaque rupture is plaque erosion (PE) [1]. 

PE recognizes a different plaque substrate as compared to plaque rupture and originates from a distinctive pathophysiological mechanism. 

In fact, PE derives from a fibrous plaque enriched by proteoglycans and hyaluronan, and with rare macrophages. Typically, the lipid component is of limited extension [2]. On the other hand, a plaque rupture typically originates from a large lipid plaque with a thin and macrophage-inflamed fibrous cap [3]. 

The pathophysiological mechanism that ends with PE is characterized by “a double hit” process. The first hit is due to disturbed local blood flow, augmented superficial shear stress leading to endothelial cell apoptosis and detachment with subsequent hyaluronan, and other extracellular matrix component exposure [4,5]. 

The second hit entails trapping of platelets after neutrophil extracellular trap (NET) formation due to activated neutrophils by toll-like receptor 2 pathway [4,6].

Yamamoto et al. [7] validated five clinical criteria that enabled the non-invasive identification of patients with ACS due to PE: hemoglobin level greater than 15 gr/dL, being younger than 68 years of age, normal renal function, evidence of anterior myocardial ischemia, and absence of diabetes mellitus [7].

The probability of an optical coherence tomography (OCT)-defined PE was as high as 73.1% when all five criteria were present in NSTE-ACS patients [7]. Although the current management of ACS patients requires urgent coronary angiography, some studies have shown that patients with PE may have a favourable prognosis compared to those presenting with plaque rupture [8,9] and that PE patients can be treated with potent antiplatelet drugs without the need of stent deployment [10].

We aimed to evaluate 20-month outcomes of ACS patients with a high probability of PE as defined by clinical criteria and those with a less likely PE, as well as admission features and laboratory results. 

## 2. Materials and Methods

### 2.1. Study Population

Patients presenting with an ACS and treated with percutaneous coronary intervention (PCI) were retrospectively enrolled between January 2015 and December 2018 at C. and G. Mazzoni Hospital, AST Ascoli Piceno, Italy. All patients were treated with second-generation drug-eluting stents during PCI. The entire ACS population included 1419 patients. Patients presenting with severe restenosis or thrombosis of a previously implanted stent as the culprit lesion or with a previous coronary artery bypass graft were excluded.

ACS patients were divided into two groups: suspected PE and less likely PE based on clinical and laboratory predictors following a previously validated approach [7].

Patients were categorized from 0 to 5 based on the number of predictors: age < 68 years old, anterior ischemia, no diabetes mellitus, hemoglobin level greater than 15 gr/dL, and normal renal function defined as an estimated glomerular filtration rate (eGFR) greater than 60 ml/min as assessed at hospital admission. Each predictor of PE was assigned point 1 if present. 

**Figure 1:**
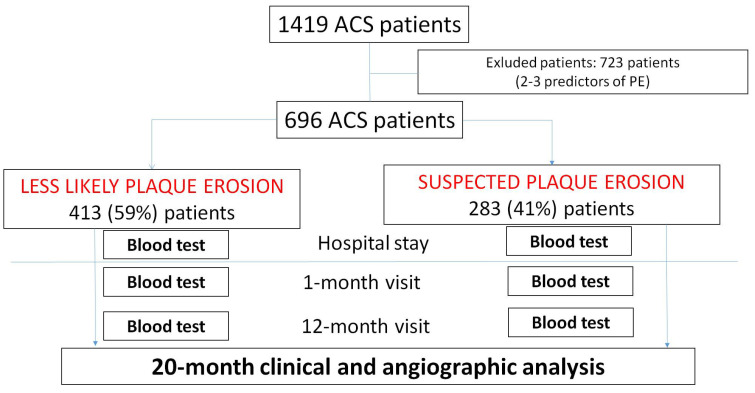
The study flow chart is presented. The entire ACS database included 1419 patients. After excluding 723 patients with 2 or 3 clinical and laboratory predictors of PE, the studied population included 696 ACS patients, which were divided into two groups (less likely plaque erosion vs. suspected plaque erosion). The studied patients underwent blood test analysis three times: at hospital admission and one month and twelve months post-hospital discharge. MACE occurrence was assessed at 20 months post-hospital discharge time.

Patients with 4 or 5 of these 5 criteria were included in the *suspected PE group*, while patients with 0 or 1 entered the *less likely PE group*. Patients with 2 and 3 criteria were not included in the comparison analysis of the two groups. A study flow chart is reported in Figure 1. In a small subset of patients, OCT imaging was performed during the index procedure at the operator’s discretion. These cases were not included in the main statistical analysis but were used for illustrative purposes.

The principles and rules of the World Medical Association Declaration of Helsinki were respected during the conduction of the study. Informed consent for research participation was obtained before coronary angiography and from identifiable subjects. Treatment decisions, including the use of ezetimibe, were left to physician discretion based on guideline-directed risk stratification and lipid targets.

### 2.2. Clinical and Coronary Angiographic Data

Cardiovascular risk factor distribution and biometric data collected at hospital admission were recorded. Coronary angiographic findings related to bifurcation involvement, the number of diseased coronary vessels, and the culprit vessel were analyzed. Medications taken by 12-month post-hospital discharge time were recorded.

### 2.3. Laboratory Data

Included patients underwent blood test analysis three times: at hospital admission and at 1-month and 12-month post-hospital discharge times.

At hospital admission, routine blood test analyses were collected as platelets, hemoglobin, glycemia, LDL cholesterol, HDL cholesterol, triglycerides, creatinine, C-reactive protein, fibrinogen, white blood cell count, peak high sensitivity troponin I, and uric acid.

At both 1-month and 12-month post-hospital discharge times, routine laboratory analyses were assessed as hemoglobin, glycemia, HDL cholesterol, LDL cholesterol, triglycerides, and creatinine. 

### 2.4. Clinical and Angiographic Endpoints

Major adverse cardiac events (MACEs) included non-fatal recurrent coronary events or death from any cause. MACEs were compared between the two groups at 20 months after hospital discharge.

A non-fatal recurrent coronary event was considered as a subsequent hospital admission for chronic coronary syndrome (CCS) or ACS requiring coronary angiography or unplanned PCI. 

ACS was defined in accordance with the Fourth Universal Definition of Myocardial Infarction [11], while CCS was defined as ischemic symptoms associated with functional or anatomical evidence of myocardial ischemia [12]. 

The non-fatal recurrent coronary event group was further divided into 3 subgroups based on angiographic findings:

(1) Target vessel revascularization (TVR) (or stent thrombosis/restenosis): if the recurrent event was secondary to significant restenosis or stent thrombosis of the stent deployed at the time of the first access for ACS. (2) Non-target vessel revascularization (non-TVR) (or non-culprit plaque progression): if the recurrent event was the consequence of progression of a previously not significant coronary plaque (luminal stenosis lower than 70% at coronary angiography) that was left untreated at the time of the first access for ACS. Lastly, (3) small vessel disease: if the recurrent event was attributed to the presence of disease of distal and small coronary vessels, it was determined that stent deployment would not be effective.

Time of recurrence was considered as the period between the first access for ACS and the subsequent unplanned hospital admission for a non-fatal recurrent coronary event. All events were independently reviewed by two experienced interventional cardiologists.

### 2.5. Statistical Analysis

The Kolmogorov–Smirnov test was used to evaluate the distribution of the data and presented as median and interquartile range or mean and standard deviation (SD) as appropriate. The Mann–Whitney U-test or the unpaired Student’s *t*-test was applied to compare continuous variables, as appropriate. Categorical variables are presented as frequencies and analyzed with the Chi-square test. The median (interquartile range) was used to describe the time interval between the first ACS episode and recurrent myocardial infarction or unplanned coronary revascularization.

The long-rank (Mantel–Cox) test was used to compare the groups, and Kaplan–Meier estimates were utilized to assess the survival curves, excluding patients who presented a MACE within the first month (to avoid a potential impact of the severity of index ACS on MACEs). 

The relationship between the suspected PE group and MACEs was assessed using Cox proportional hazards models. Two models were developed to adjust for confounders: Model 1 (baseline model, unadjusted) and Model 2, which was adjusted for relevant residual risk determinants [13,14,15,16,17,18,19,20] such as age, sex, diabetes mellitus, chronic kidney disease (defined as an eGFR lower than 60 ml/min), and non-ST-segment elevation myocardial infarction (NSTEMI) as a type of ACS presentation. Hazard ratios (HR) and 95% confidence intervals (CI) for the covariates were calculated.

A *p*-value < 0.05 was considered significant.

Statistical analysis was performed using SPSS 21.0 (SPSS, Inc., Chicago, IL, USA).

## 3. Results

### 3.1. Clinical and Angiographic Characteristics of the Included Population

The study included 696 ACS patients. The suspected PE group was composed of 283 (41%) patients, while the less likely PE group consists of 413 (59%) patients. 

Among the 1419 ACS patients initially evaluated, the proportion of patients with suspected PE was approximately 20% (283/1419), when accounting for those excluded due to having 2–3 PE predictors.

Baseline clinical characteristics of the entire population and the two groups are shown in Table 1.

The mean age of the entire studied population was 70.1 years ± 13, and the mean body mass index (BMI) was 26.9 ± 4.4. Seventy-two percent of patients were male, and one diseased coronary vessel was observed in 38%. Diabetes mellitus was observed in 49.5% of patients and chronic kidney disease in 37.9%.

The suspected PE group had a lower mean age, a higher frequency of male sex, and a higher BMI. Current smokers were more commonly seen in suspected PE as compared to the less likely PE group. 

The 723 excluded patients with 2 or 3 PE predictors had intermediate baseline characteristics, including a mean age of 66.3 ± 11.5 years, 65% male sex, 39% diabetes, and 28% chronic kidney disease. These values were between those observed in the suspected and less likely PE groups.

Single vessel disease was observed in 50% of the suspected PE group as compared to 30% of the less likely PE group (*p* = 0.0001). Table 2.

The left anterior descending artery was the culprit vessel in 76% of suspected PE patients as compared to 24% of less likely PE patients (*p* = 0.0001). At the 12-month follow-up time, suspected PE patients were more on ezetimibe as compared to the less likely PE patients (41% and 19%, respectively, *p* = 0.0001). No significant differences were found for statin, potent P2Y12 inhibitors (ticagrelor or prasugrel use), and beyond 1-year dual antiplatelet therapy (Table 3).

### 3.2. Laboratory Data at Hospital Admission and 1-Month, and 12-Month Post-Hospital Discharge Times

Laboratory results obtained at hospital admission are represented in Table 4, while laboratory analyses collected at 1-month and 12-month post-hospital discharge times are presented in Table 5.

At baseline, C-reactive protein and fibrinogen were significantly lower in the suspected PE group as compared to the less likely PE one. Both LDL and triglycerides were significantly higher in the suspected PE group as compared to the less likely PE one, as well as white blood cell count. 

At both 1-month and 12-month post-hospital discharge, LDL cholesterol and triglycerides were not significantly different between the two groups. Hemoglobin level remained significantly higher, while creatinine and glycemia were significantly lower in the suspected PE group as compared to the less likely PE one. 

### 3.3. Major Adverse Cardiovascular Events in the Two Groups at the 20-Month Time

**Figure 2:**
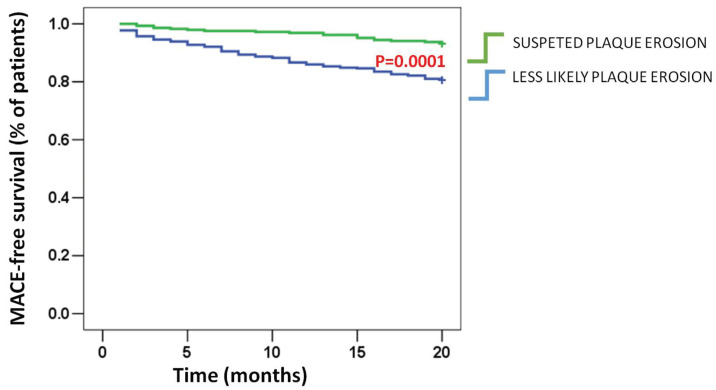
Kaplan–Meier curves for MACEs at 20-month follow-up time. MACE event-free survival Kaplan–Meier curves in the less likely PE vs. suspected PE groups are shown. Data are assessed throughout the 20-month post-hospital discharge time and analyzed by applying the log-rank test.

The suspected PE group had a significantly lower MACE rate at 20-month post-discharge time as compared to the less likely PE one (7.4% as compared to 28.8%, *p* = 0.0001) (Table 6 and Figure 2). 

Multivariate Cox regression models were constructed to analyze the association between the suspected plaque erosion group and MACEs (Table 7).

In Model 1 (unadjusted), the presence of the suspected PE group was significantly associated with a reduced risk of MACEs (HR = 0.20, 95% CI: 0.12–0.35, *p* = 0.0001). Model 2 was adjusted for covariates, and the results were similar, showing that patients with suspected plaque erosion had a lower risk of MACEs (HR = 0.37, 95% CI: 0.18–0.76, *p* = 0.007). 

Death from any cause was significantly lower in the suspected PE group as compared to the less likely PE one (2.1% as compared to 24.9%, *p* = 0.0001). 

**Figure 3:**
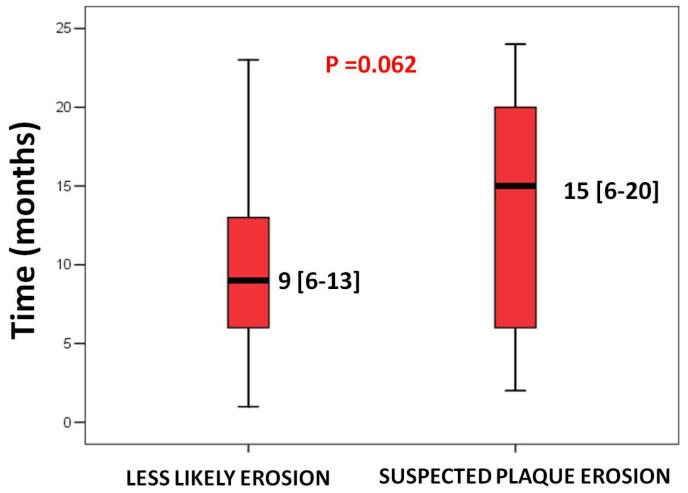
Box-and-whisker plots representing the time of recurrence comparing the less likely PE vs. suspected PE groups.

Non-fatal recurrent coronary events were not statistically different between the two groups, although the median time interval between the first ACS episode and recurrent myocardial infarction or unplanned coronary revascularization tended to be longer in the suspected PE group as compared to the less likely PE group (15 months (6–20) vs. 9 months (6–13), *p* = 0.062) (Figure 3).

Time of recurrence is calculated as the number of months from the index procedure to the recurrent non-fatal coronary event date and expressed as a median and interquartile range. 

No significant differences were seen for the rate of TVR, non-TVR, and small vessel coronary disease between the two groups (Table 8).

Similarly, the rate of ACS and CCS as clinical presentations of the recurrent coronary event were similar between the suspected PE group and the less likely PE one (Table 9). 

## 4. Discussion

The present study identified four main results:

The prevalence of suspected PE was 20% in a cohort of ACS patients using the reported clinical and laboratory criteria for PE identification and including patients from 0 to 5 predictors of PE.

Suspected PE patients were more frequently current smokers and had higher BMI. 

Lower levels of both CRP and fibrinogen, together with a higher prevalence of single-vessel disease, were more commonly seen in suspected PE.

Suspected PE patients showed a lower rate of MACEs at 20 months, largely due to the lower mortality rate from any cause. 

### 4.1. Prevalence of Suspected PE Among ACS Patients

In post-mortem examinations of individuals who died of myocardial infarction, the prevalence of PE as the underlying pathology has ranged between 25% and 40% [1]. Jia et al. reported an in vivo incidence of PE at 31% in a group of ACS patients utilizing OCT [2]. Currently, an in vivo diagnosis of PE remains a diagnosis of exclusion [21]. OCT studies have shown a different prevalence of plaque erosion across ACS, being more common in NSTEMI (47.9%) and less common in patients with ST-segment elevation myocardial infarction (STEMI) (29.8%) [1,4].

We showed a prevalence of suspected plaque erosion of 20% by applying clinical criteria for the PE identification in the entire cohort of ACS patients. This lower proportion of suspected PE (20%) compared to previous reports may be due to the strict criteria used to define both comparison groups and the presence of a large intermediate group with 2–3 predictors. Those patients with 2–3 predictors may represent a heterogeneous population in which the likelihood of PE is uncertain. These patients were therefore not included in the subsequent comparisons of extreme groups.

Among 401 patients with STEMI, 166 (41.4%) met 4 or 5 predictors of PE and were classified as suspected PE. Among the 295 NSTEMI patients, 117 (39.7%) fulfilled 4 or 5 criteria. The actual prevalence of plaque erosion in these subgroups could not be determined due to the absence of systematic OCT imaging, and the predictive performance of these criteria in STEMI remains uncertain.

This study applied clinical and laboratory criteria to identify suspected PE subjects.

These clinical/laboratory criteria were originally validated against OCT predominantly in NSTE-ACS cohorts [7]; in the present study, we applied them to the entire ACS population (STEMI and NSTEMI) to compare groups with a high versus a low likelihood of PE based on non-invasive features. However, an in vivo OCT study reported that younger age, anterior infarction/LAD culprit, and certain laboratory profiles are associated with erosion even in STEMI patients [22]. Thus, we may speculate that the predictors of PE can also be considered in STEMI patients

Indeed, OCT is the gold standard to identify PE in ACS patients. In fact, OCT enables a detailed image of the coronary plaque, allowing precise identification of plaque features and thrombus composition. In addition, recent studies indicate that even pre-procedural computed tomography angiography may assist in predicting OCT-defined PE [23]. These imaging modalities were not accessible to all patients within this retrospective study, constraining our ability to validate clinical criteria by direct visualization.

### 4.2. Cardiovascular Risk Factor Distribution in Suspected PE Patients

Early autopsy studies showed an association between smoking and PE in premenopausal women who were victims of sudden cardiac death [24,25]. However, in vivo studies reported unequivocal results, such as the results by Ferrante et al., who showed a significant association between smoking and plaque erosion [26], by Seegers et al., who showed an increased risk of PE only in male smoker patients instead of women smokers [27], and by Abtahian et al., who showed an increased association between current smokers and the risk of plaque rupture [28]. Part of this discrepancy may derive from the clinical presentations of the enrolled population as well as the average age of the included subjects. Dai et al. investigated a STEMI population with a mean age of 57.7 years, which is very similar to the one of our suspected PE group, and observed that current smoking was significantly associated with PE [22]. 

Previous studies showed that patients with PE have a lower prevalence of traditional cardiovascular risk factors such as dyslipidemia [29] as well as a better lipid profile [22,30]. In our study, we did not find a significant difference between the suspected PE and the less likely PE groups in terms of dyslipidemia at hospital admission (51% vs. 48%, *p* = 0.488). However, both LDL cholesterol and triglycerides were higher at admission. 

We also identified a slightly, but significantly, higher BMI in the suspected PE group as compared to the less likely one (27.6 vs. 26.4, *p*= 0.0001). Other studies did not find differences in terms of obesity as defined as a BMI greater than 25 [30] or 30 [9].

Indeed, a previous study showed that LDL cholesterol was higher in PE patients presenting with STEMI as compared to NSTEMI [7]. Since in our cohort studied ACS population, the STEMI cohort (59%) was greater than the NSTEMI one (41%), we may speculate that the worst lipid profile and the higher BMI seen in our suspected PE group may derive from the higher frequency of STEMI presentation. Interestingly, both LDL cholesterol and triglycerides were not different between the two groups at 1-month and 12-month post-discharge times, probably because there was a higher rate of ezetimibe usage at 12-month post-discharge in the suspected PE group (41.7% vs. 19.1%, *p* = 0.0001). Differences in ezetimibe use at follow-up reflect physician-driven decisions and may have contributed in part to the observed differences in long-term outcomes.

### 4.3. Systemic Inflammation and Coronary Atherosclerosis Burden in Suspected PE Group

Lower levels of both C-reactive protein and fibrinogen, together with a higher frequency of one diseased coronary vessel, were seen in suspected PE. These data are in line with previous studies that showed PE associated with less systemic inflammation [31] and lower overall plaque burden as compared with plaque rupture [32]. 

Another study also showed that not only systemic inflammation but also coronary vascular inflammation was lower in PE when assessed by pericoronary adipose tissue attenuation [23].

This data supports a distinctive pathophysiological mechanism involved in PE. This mechanism requires recruitment and activation of neutrophils rather than monocytes and macrophages, as seen in plaque rupture. The level of systemic inflammatory reaction associated with neutrophil activation/recruitment is much lower and typically involves specific biomarkers such as myeloperoxidase [26,33] rather than C-reactive protein.

This lower degree of systemic inflammation is also associated with fewer features of plaque vulnerability at both the culprit site and remote coronary segments [34]. 

**Figure 4:**
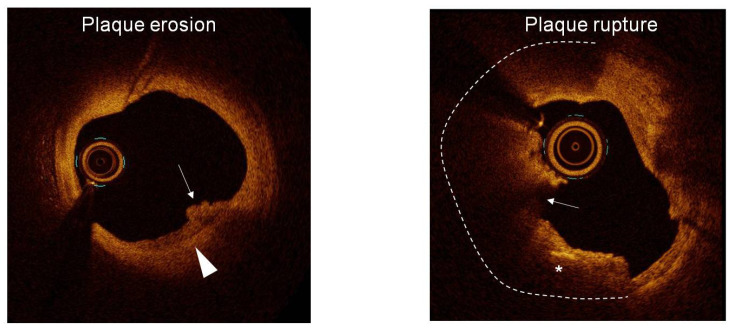
Representative OCT images from two different patients enrolled in the study, showing plaque erosion and plaque rupture. Plaque erosion is imaged as a white (platelet-enriched) thrombus (arrow) over a fibrous plaque with an intact and thick fibrous cap (arrowhead). Plaque rupture is shown as a fibrous cap discontinuity associated with red (erythrocyte-enriched) thrombus. The underlying plaque is a lipid plaque (dashed line), and a cholesterol crystal is also imaged in the context of the lipid plaque (asterisk).

PE is typically observed as a complication of a fibrous plaque or lipid plaque with a limited extension of lipid material. The fibrous cap thickness was also shown to be higher in PE as compared to plaque rupture. The prevalence of thin-cap fibroatheroma was only 10.3% in PE as compared to 67.3% in plaque rupture [2] (Figure 4).

A previous study showed that PE was associated with less calcification and thrombus burden as well as lower prevalence of complex lesions [28] at the culprit site. This data suggests a less advanced and more focal atherosclerosis in cases of PE [3]. 

This limited extension of the atherosclerotic process is also seen in remote regions, suggesting a lower pancoronary vascular vulnerability in patients presenting with PE. Sugiyama et al. showed that patients presenting a PE as the culprit lesion were characterized by a lower number of non-culprit plaques in remote coronary segments, and that these plaques had less frequent signs of vulnerability, such as micro-vessels and macrophages as compared to plaque rupture [31]. 

### 4.4. Suspected PE Patients Have a Better Prognosis: The Role of Tailored Treatment for PE Management

Previous studies showed that patients with PE have a better prognosis as compared to plaque rupture [9,30,35]. 

We confirmed that the suspected PE group had a significantly lower rate of MACEs at the 20-month follow-up (7.4% vs. 28.8%, *p* = 0.0001). Even after adjusting for potential confounders, the suspected PE group remained strongly associated with a lower risk of MACEs. 

Even though multivariable adjustment was conducted, there may still be some confounding factors. 

In addition, the cause of death (cardiac vs. non-cardiac) was not systematically recorded; therefore, we included all-cause mortality in the composite MACE definition to ensure completeness of outcome data. We recognize that this approach may reduce specificity for coronary outcomes, especially considering the older age and higher comorbidity burden in the less likely PE group. Therefore, the present results should be considered as associative rather than causal. We also showed that death from any cause was significantly lower in the suspected PE group (2.1% vs. 24.9%, *p* = 0.0001). 

The observed difference in all-cause mortality between the suspected and less likely PE groups appears to reflect the markedly different baseline clinical profiles of the two groups. In particular, the less likely PE group was significantly older and had a higher burden of comorbidities such as diabetes mellitus and chronic kidney disease, both of which are strong predictors of long-term mortality in ACS patients. Importantly, cause-specific mortality data were not available, and our findings refer to all-cause mortality. The lack of significant differences in recurrent coronary events may reflect the broader impact of improved secondary prevention strategies over the past decade, which have contributed to lower event recurrence rates across a range of patient populations [19,20], including elderly ACS patients [36].

Our results are in line with the ones by Yonetsu et al. [35], who showed a lower MACEs rate in OCT-defined PE patients as compared to plaque rupture. However, they showed a significant reduction in terms of non-fatal recurrent coronary events. 

We failed to identify a significant difference in terms of the non-fatal recurrent coronary event rate.

We may speculate that although the two studies had a similar follow-up time (20 months), the overall rate of non-fatal recurrent coronary events was lower in our study. A possible explanation is that we included all ACS patients, while Yonetsu et al. [35] excluded patients in cardiogenic shock and patients with renal insufficiency. We also may speculate that the overall treatment and targets for ACS patients have been changed between the two studies and the rate of recurrent coronary events is currently lower as compared to the past [37].

Contrary to our findings, Yang et al. [38] reported improved outcomes in patients with plaque rupture. This discrepancy may derive from differences in patient selection, use of intracoronary imaging for plaque characterization, and clinical management. Importantly, the suspected PE group had fewer comorbidities and less severe coronary artery disease, which may have affected the results.

A previous study of our group with a longer follow-up time (30 months) showed a lower rate of MACEs in the PE group, mainly due to the reduced rate of non-TVR in PE patients (12% vs. 3.5%, *p* = 0.07) [9]. In the present study, conducted several years later than the first, we identified a huge reduction in overall non-TVR rate, being 1.7% as compared to 12%, probably due to better management of ACS patients. In the present study, we did not find a significant difference in terms of non-TVR in the two groups, possibly due to exceptionally low results observed in both groups (1% vs. 2.1%, respectively).

The better long-term prognosis observed in the suspected PE group, despite their higher baseline LDL-C and triglyceride levels, may be explained by the group’s younger age, lower inflammatory burden, and lower prevalence of high-risk comorbidities such as diabetes mellitus and chronic kidney disease. Additionally, the more frequent use of ezetimibe at 12-month follow-up suggests a more intensive lipid-lowering strategy over time. It is also worth noting that baseline lipid levels at ACS presentation may not fully reflect long-term lipid control, particularly in the context of aggressive secondary prevention. Moreover, plaque vulnerability and outcomes may be more closely linked to plaque composition and systemic inflammation than to isolated lipid values [39].

We found that the median time interval between the index ACS episode and the recurrent episode tended to be longer in the suspected PE group as compared to the less likely PE group (15 months (9–20)vs. 9 months (6–13) *p* = 0.062). This result may be explained by the higher incidence of post-stenting complications seen in cases of plaque rupture. In fact, Hu et al. showed that a higher frequency of in-stent dissection, stent malapposition, and thrombus after stenting plaque ruptures, and this higher complication rate may justify the early recurrence time [30]. 

## 5. Conclusions

The present study confirmed the role of non-invasive and clinically based assessment of patients suspected of having PE. We further validated clinical predictors of PE, showing that patients with suspected PE have a lower grade of systemic inflammation and a reduced overall plaque burden. In our cohort, the suspected PE group had a better prognosis at 20 months, driven by lower all-cause mortality; however, this observation may be related to their more favorable baseline clinical profile, and no causal relationship can be inferred. A low rate of both TVR and non-TVR events has been observed in the present study. 

These results, together with evidence from recent studies [4,28], support the concept of tailoring the management of ACS patients with suspected PE. Prior studies have suggested that, in selected STEMI patients with PE as the culprit lesion, stable coronary flow and non-significant residual stenosis after initial PCI may be managed without immediate stent deployment [40,41].

### Limitations

The present study has several limitations.

Firstly, we categorized ACS patients into two groups: suspected PE and the less likely plaque erosion based on the previously published clinical and laboratory criteria. 

The clinical and laboratory predictors of PE were primarily validated in NSTE-ACS; their predictive performance in STEMI within our cohort was not formally assessed, and our comparisons should be interpreted as associative.

Our choice to exclude patients with 2–3 PE predictors was aimed at enhancing the difference between groups, but it might have introduced a selection bias. Thus, we cannot extend our results to the entire ACS population, since patients with intermediate probability of PE represent a large portion of it.

We did not confirm the diagnosis of PE by intravascular imaging. While this may reduce diagnostic specificity, such an approach reflects real-world constraints and is supported by the literature identifying robust clinical correlates of PE. Although based on criteria previously compared to OCT [7], the absence of imaging confirmation in our cohort presents a risk of misclassification bias, as highlighted by Partida et al. [4,5]. This limitation must be considered when interpreting our findings.

Ongoing efforts are refining multi-parametric algorithms to enhance diagnostic accuracy in patients not undergoing invasive imaging.

Secondly, since both diabetes mellitus and chronic kidney disease were two of these criteria, we observed exceptionally low prevalence of both diseases in the suspected PE groups (Table 1). The low prevalence of the diseases within the younger age bracket may have impacted the lower rate of death from any cause, as seen in suspected PE groups.

Thirdly, all the included ACS patients underwent second-generation stent deployment. As a result, we cannot generalize these findings to PE patients who were medically managed as part of the EROSION trial [10]. Treatment strategies were not standardized or protocol-driven, and variation in follow-up lipid-lowering therapy may have influenced clinical outcomes.

In addition, the study lacked some data on key clinical severity markers at the time of ACS presentation, such as Killip class, GRACE risk score, left ventricular ejection fraction (LVEF), and infarct size. These variables were not systematically collected in our historical dataset and therefore could not be included in the multivariable models. Their absence may represent a source of residual confounding in the interpretation of our prognostic comparisons.

Lastly, the suspected PE group had a lower incidence of MACEs, driven by a higher rate of death from any cause. The rate of non-fatal RCEs was not different. However, the suspected PE group had a better clinical profile at admission since they were younger and had a lower incidence of diabetes mellitus and chronic kidney disease. Cox proportional hazards models, applied for accounting for confounders, confirmed a lower risk of MACEs in the suspected PE group. However, we cannot exclude that the clinical criteria applied for identifying both suspected PE and the less likely PE groups may have selected a restricted cohort of patients. As a result, we are unable to generalize the current findings to a wider ACS population.

## Figures and Tables

**Table 1:** Baseline clinical characteristics in the overall population and stratified by PE group.

	Overall (696 Patients)	Less Likely Plaque Erosion (413 Patients)	Suspected Plaque Erosion (283 Patients)	*p* Value
Age, years	70.1 ± 13.4	79.0 ± 7.5	57.2 ± 8.8	0.0001
Male sex, *n* (%)	501 (72)	241 (58)	260 (91.9)	0.0001
Weight, Kg	76.4 ± 15.3	71.9 ± 13.9	83.0 ± 15.0	0.0001
Height, cm	168 ± 8.9	164.6 ± 8.3	172.9 ± 7.2	0.0001
BMI, Kg/m^2^	26.9 ± 4.4	26.4 ± 4.4	27.6 ± 4.2	0.0001
Smoker				
Never, *n* (%)	418 (60)	272 (66)	146 (52)	0.0001
Past, *n* (%)	153 (22)	95 (23)	58 (21)	
Current, *n* (%)	125 (18)	46 (11)	79 (28)	
Dyslipidemia, *n* (%)	343 (49)	199 (48)	144 (51)	0.488
Diabetes mellitus, *n* (%)	345 (49.5)	296 (71.6)	49 (17.3)	0.0001
Chronic kidney disease, *n* (%)	264 (37.9)	219 (53)	45 (15.9)	0.0001
STEMI, *n* (%)	401 (57)	235 (57)	166 (59)	0.696
NSTEMI, *n* (%)	295 (43)	178 (43)	117 (41)	

Values are expressed as mean (standard deviation) or absolute number of cases (relative percentage) as appropriate. BMI: body mass index, STEMI: ST-segment elevation myocardial infarction, NSTEMI: non-ST-segment elevation myocardial infarction.

**Table 2:** Coronary angiographic characteristics in the overall population and stratified by PE group.

	Overall (696 Patients)	Less Likely Plaque Erosion (413 Patients)	Suspected Plaque Erosion (283 Patients)	*p* Value
Culprit vessel				0.0001
Left main, *n* (%)	14 (2)	12 (3)	2 (1)	
Left anterior descending artery, *n* (%)	314 (45)	99 (24)	215 (76)	
Circumflex, *n* (%)	154 (22)	127 (31)	27 (9)	
Right coronary artery, *n* (%)	214 (31)	172 (42)	42 (15)	
One-vessel disease, *n* (%)	264 (38)	123 (30)	141 (50)	0.0001
Three-vessel disease, *n* (%)	139 (20)	103 (25)	36 (13)	0.004
Coronary bifurcation involvement, *n* (%)	193 (28)	77 (19)	116 (41)	0.0001

Values are expressed as the absolute number of cases (relative percentage).

**Table 3:** Pharmacological treatment at 12-month follow-up in the overall population and stratified by PE group.

	Overall (698 Patients)	Less Likely Plaque Erosion (413 Patients)	Suspected Plaque Erosion (283 Patients)	*p* Value
Clopidogrel, *n* (%)	318 (45.7)	188 (45.5)	130 (45.9)	0.938
Potent P2Y12 inhibitor, *n* (%)	417 (59.9)	255 (61.7)	162 (57.2)	0.239
DAPT > 1 year, *n* (%)	113 (16.2)	74 (17.9)	39 (13.8)	0.174
Ezetimibe, *n* (%)	197 (28.3)	79 (19.1)	118 (41.7)	0.0001
High-intensity statin, *n* (%)	638 (91.4)	377 (90.8)	261 (92.2)	0.583

Values are expressed as the absolute number of cases (relative percentage). DAPT: dual antiplatelet treatment.

**Table 4:** Laboratory parameters at hospital admission in the overall population and stratified by PE group.

	Overall (696 Patients)	Less Likely Plaque Erosion (413 Patients)	Suspected Plaque Erosion (283 Patients)	Overall (696 Patients)
Hemoglobin, gr/dL	13.6 ± 2.1	12.4 ± 1.7	15.2 ± 1.3	0.0001
White blood cells, 1000 xmm^3^	10.2 ± 4.6	9.8 ± 4.0	10.8 ± 5.3	0.012
C-Reactive Protein, mg/dL	3.6 ± 5.8	4.4 ± 6.4	2.7 ± 4.7	0.017
Fibrinogen, mg/dL	486.9 ± 172.2	516.4 ± 166.1	446.6 ± 159.7	0.024
High sensitivity troponin I, ng/L	2482.6 ± 2863.4	2473.0 ± 2825.1	2489.2 ± 2894.5	0.953
Platelets, 1000/µl	227.4 ± 72.6	223.5 ± 79.7	232 ± 61.4	0.110
Uric acid, mg/dL	6.2 ± 1.7	6.3 ± 1.9	6.1 ± 1.5	0.234
LDL cholesterol, mg/dL	122.1 ± 38.1	108.1 ± 33.3	139.7 ± 36.6	0.0001
HDL cholesterol, mg/dL	42.1 ± 9.7	42.2 ± 8.8	42.2 ± 8.8	0.766
Triglyceride, mg/dL	134.7 ± 72.3	126.6 ± 58.8	145.2 ± 85.8	0.002
Creatinine, mg/dL	1.2 ± 0.9	1.4 ± 1.1	0.9 ± 0.2	0.0001
Glycemia, mg/dL	138.3 ± 64.5	161.7 ± 68.6	117.3 ± 52.3	0.0001

Values are expressed as mean (standard deviation).

**Table 5:** Laboratory parameters at 1-month and 12-month follow-up in the overall population and stratified by PE group.

	Overall (696 Patients)	Less Likely Plaque Erosion (413 Patients)	Suspected Plaque Erosion (283 Patients)	*p*-Value
At 1-month follow-up				
Creatinine, mg/dL	1.1 ± 0.7	1.3 ± 0.9	0.9 ± 0.1	0.0001
Glycemia, mg/dL	113.1 ± 35.3	123.4 ± 42.9	101.9 ± 19.1	0.0001
HbA1c, %	6.8 ± 1.2	7.2 ± 1.1	5.9 ± 0.9	0.0001
Hemoglobin, gr/dL	13.5 ± 1.9	12.4 ± 1.7	14.7 ± 1.2	0.0001
LDL cholesterol, mg/dL	79.1 ± 27.7	79.0 ± 30.2	79.2 ± 25.1	0.961
HDL cholesterol, mg/dL	42.9 ± 11.3	44.4 ± 9.5	43.3 ± 12.9	0.500
Triglycerides, mg/dL	126.0 ± 60.4	122.5 ± 54.1	129.3 ± 65.9	0.380
At 12-month follow-up				
Creatinine, mg/dL	1.3 ± 3.1	1.7 ± 4.4	0.9 ± 0.2	0.048
Glycemia, mg/dL	118.4 ± 41.5	133.4 ± 52.0	102.5 ± 14.3	0.0001
HbA1c, %	6.6 ± 1.1	7.2 ± 1.0	5.7 ± 0.4	0.0001
Hemoglobin, gr/dL	13.5 ± 1.9	12.4 ± 1.7	14.7 ± 1.2	0.0001
LDL cholesterol, mg/dL	75.2 ± 26.9	74.4 ± 26.8	76.0 ± 27.1	0.636
HDL cholesterol, mg/dL	43.8 ± 10.3	43.9 ± 10.3	43.7 ± 10.3	0.899
Triglycerides, mg/dL	129.6 ± 66.8	127.1 ± 68.5	132.3 ± 65.2	0.616

Values are expressed as mean (standard deviation).

**Table 6:** Incidence of major adverse cardiac events (MACE) at 20-month follow-up in the overall population and stratified by PE group.

	Overall (696 Patients)	Less Likely Plaque Erosion (413 Patients)	Suspected Plaque Erosion (283 Patients)	*p* Value
MACE, *n* (%)	140 (20.1)	119(28.8)	21 (7.4)	0.0001
Death from any cause	109 (15.7)	103(24.9)	6 (2.1)	0.0001
Non-fatal recurrent coronary event, *n* (%)	41 (5.9)	26 (6.3)	15 (5.3)	0.626

Values are expressed as the absolute number of cases (relative percentage). MACE: major adverse coronary event.

**Table 7:** Multivariable Cox regression analysis for predictors of MACEs at 20 months.

	HR (95% CI)	*p* Value
Suspected plaque erosion	0.37 (0.18–0.76)	0.007
NSTEMI as a type of ACS presentation	1.32 (0.89–1.96)	0.162
Chronic kidney disease	0.79 (0.54–1.17)	0.253
Diabetes mellitus	0.95 (0.65–1.38)	0.782
Male sex	0.91 (0.63–1.32)	0.645
Age	1.02 (1.01–1.05)	0.031

Model 2 adjusted for NSTEMI presentation, chronic kidney disease, diabetes mellitus, male sex, and age.

**Table 8:** Characteristics of recurrent coronary events in the overall population and stratified by PE group.

	Overall (696 Patients)	Less Likely Plaque Erosion (413 Patients)	Suspected Plaque Erosion (283 Patients)	*p*-Value
Non-TVR, *n* (%)	12 (1.7)	9 (2.1)	3 (1.0)	0.608
TVR, *n* (%)	19 (2.7)	11 (2.6)	8 (2.8)	
Small vessel disease, *n* (%)	10 (1.4)	6 (1.4)	4 (1.4)	

Values are expressed as the absolute number of cases (relative percentage). TVR: target vessel revascularization.

**Table 9:** Clinical presentation of recurrent coronary events (ACS vs. CCS) in the overall population and stratified by PE group.

	Overall (41 RCES)	Less Likely Plaque Erosion (26 RCEs)	Suspected Plaque Erosion (15 RCEs)	*p*-Value
Chronic coronary syndrome, *n* (%)	9 (22)	4 (15.4)	5(33.3)	0.248
Acute coronary syndrome, *n* (%)	32 (78)	22 (84.6)	10 (66.7)	

Values are expressed as the absolute number of cases (relative percentage). RCEs: recurrent coronary events.

## Data Availability

The data underlying this article will be shared on reasonable request to the corresponding author.

## Abbreviations

The following abbreviations are used in this manuscript:

ACS	acute coronary syndromes
BMI	body mass index.
CCS	chronic coronary syndrome
eGFR	estimated glomerular filtration rate
HR	hazard ratios.
MACEs	major adverse cardiac events
NET	neutrophil extracellular trap
non-TVR	non-target vessel revascularization
NSTEMI	non-ST-segment elevation myocardial infarction
OCT	optical coherence tomography
PCI	percutaneous coronary intervention
PE	plaque erosion
SD	standard deviation
STEMI	ST-segment elevation myocardial infarction
TVR	target vessel revascularization
